# Supplementary material for: BOLD and GenBank revisited – Do identification errors arise in the lab or in the sequence libraries?
Source: PLoS One. 2020 Apr 16;15(4):e0231814. doi: 10.1371/journal.pone.0231814 (PMC7162515; doi:10.1371/journal.pone.0231814)
Supplement: Attachment — Submitted filename: Response to Reviewers.docx [file pone.0231814.s003.docx]

To the editor:

We thank the reviewers for scrutinizing our manuscript and providing constructive feedback. We agree with the suggestions made by Reviewer 2, and have edited the manuscript text and Figure 2 accordingly. We have also revised the formatting of the manuscript file to comply with the PLOS style requirements as requested by the editor. All changes are documented in the annotated version of the revised manuscript file.

Sincerely,

Mikko Pentinsaari

Sujeevan Ratnasingham

Scott E. Miller

Paul D. N. Hebert
