# Supplementary material for: BOLD and GenBank revisited – Do identification errors arise in the lab or in the sequence libraries?
Source: PLoS One. 2020 Apr 16;15(4):e0231814. doi: 10.1371/journal.pone.0231814 (PMC7162515; doi:10.1371/journal.pone.0231814)
Supplement: Attachment — Submitted filename: PONE-D-19-20600R1_Comments to Authors.docx [file pone.0231814.s004.docx]

**­­Review of:** ***Forensics and DNA barcodes – do identification errors arise in the lab or in the sequence libraries?***

**PONE-D-19-20600R1**

**Paper summary:**

This paper seeks to identify reasons why the results presented in Meiklejohn et al (PLoS One. 2019;14: e0217084. Doi:10.1371/journal.pone.0217084) provide a misleading evaluation of the data contained in BOLD. In Meiklejohn et al, appropriate DNA barcode sequences from 17 insect, 61 plant and 16 macro-fungi species (curated reference material sourced from national collections) were obtained and compared to both GenBank and BOLD. Specifically for insect taxa, Meiklejohn et al reported that GenBank out performed BOLD with respect to correct species level identifications. In the paper under review, the authors identify three main reasons for the misidentifications reported in Meiklejohn et al using BOLD: 1) reverse complement sequences were searched against BOLD, 2) sequences were cross-contaminated, and 3) taxa included were either misidentified or part of a species complex.

**Major Technical Comments:**

**1) Line 24-26:** The authors made the claim that they have accounted for all of the misidentifications in Meiklejohn et al. by identifying errors in the methods (*i.e.,* “*problems in the reference libraries or in the query sequences used to test them*”). This idea is later contradicted in line 48 when they restated this as “..*most, if not all, of the misidentifications*” and later in line 82-83 where the authors implied that misidentifications also occurred due to “*errors in the reference libraries..*”, which was one of the primary conclusions of Meiklejohn et al. The authors contradicted themselves throughout their manuscript and need to update the text for consistency (based on valid data and conclusions – discussed in later points).

**2) Line 41-42:** Authors provided the statement, “*Because COI generally has high accuracy in species assignment [4–9], the conclusions from a recent study by Meiklejohn et al. [10] were surprising*.”. This statement would lead readers to believe that Meiklejohn et al did not acknowledge that their results did not conform to the majority of previous studies examining the accuracy of BOLD. This is not the case, as Meiklejohn et al. highlighted this by stating “*This result was lower than previously reported for flies (Diptera [15,20], beetles (Coleoptera [23]), butterflies and moths (Lepidoptera [24]), when searching against either or both of these databases*.” Thus, the text needs to be modified to reflect that Meiklejohn et al. also reported that their results were lower than previously reported.

**3) Line 71-75:** It is apparent that Meiklejohn et al did indeed search the reverse complement for seven COI sequences against BOLD. In doing so, the matches they returned were lower with respect to the similarity statistic and were to the incorrect species (highlighted by the authors in Table 1). While it is good scientific practice to search sequences in the forward orientation, at the time in which Meiklejohn et al searched sequences against BOLD (reported in their paper as “July 2017-January 2018”), there was no guidance or requirement that COI sequences must be searched in the forward orientation; neither Ratnasingham and Hebert (2007) nor the “BOLD Print Handbook for BOLD (v3 or v4)” specified the orientation of query sequences. Further, it is apparent the identification interface for COI on boldsystems.org was updated only ~6 months ago (after Meiklejohn et al was published) to state “*Enter fasta formatted sequences in the forward orientation*” and if a reverse complement sequence is submitted the result states “*These results may be due to reverse or reverse complement sequences. Please try again with the forward orientation”.* Conversely, the BOLD database always accommodated both orientations for plants and fungal identification engines. Thus, when researchers completed the searches against the original BOLD, they would have had no reason to immediately assume that the incorrect matches were a result of query sequence orientation. While the authors are correct in identifying a source of error in Meiklejohn et al, they have neglected to address why this mistake might have occurred. Given it was not previously explicitly stated that forward sequences must be searched (solely for insect COI sequences; not fungal or plant sequences), it is possible that results from other researchers might also have been affected by this issue and so must be addressed in this manuscript.

Meiklejohn et al. wrote that the BOLD similarity metric was an indicator of an incorrect match and recommended that it is important to use the metric to determine confidence of the results. In Meiklejohn et al., results that gave lower similarity were not excluded, presumably in order to see what the database results gave as the “most similar match”.

**4) Line 89-91:** The authors made a bold statement and suggested that “*misidentification clearly arose from contamination*” and made a reasonable hypothesis as to a potential cause for misidentifications. However, they failed to meet their burden of proof as well as contradicted themselves later in the article. In line 85-87, the authors admitted that “*the other three mismatches involve taxa unrepresented among the 17 tested species ruling out internal contamination.*” If internal contamination were the cause of one sample’s misidentification, then the authors need to provide an explanation for the lack of this contamination in the other samples. It is at this point that the authors contradicted themselves and by writing, “*This leaves two possible explanations-contamination in the reference sequences libraries or in the query sequences.”* Again, the authors have not met their burden of proof for either claim of contamination. Furthermore, misidentification caused by inconsistencies in reference sequence libraries was a major conclusion cited by Meiklejohn et al. as a source for misidentification in the first place. Furthermore, Meiklejohn et al. used controls during sample processing, a procedure that was referenced in their material and methods (see further explanation below). Even after making these concessions, the authors in line 91 wrote, “*these cases of misidentification clearly arose from contamination of query sequences*,” again, contradicting themselves.

For the four specimens in which the COI sequences were identified by the authors as stemming from cross-contamination, only one of those sequences corresponded to a species processed in Meiklejohn et al (*Hexagenia limbata*). Typically, inadvertent cross-contamination arises when a laboratory routinely processes large numbers of similar samples. Do the authors know whether the research division of the FBI Laboratory (where Meiklejohn et al performed the research) processes large numbers of insect material? If so, they should state that as a plausible source of DNA for cross-contamination, rather than just providing a broad sweeping explanation of “cross-contamination”.

Overall for this section (subheading “Factors responsible for four ‘errors’ in generic assignment”) the authors need to make clear and well-supported arguments, rather than swapping between possible explanations for the misidentifications and contradicting themselves. Substantial modifications to the text are needed to address this.

**5) Line 91-94:** Authors wrote, “*Cross-contamination is a well- recognized risk when working with museum specimens, so it is standard practice to check for its occurrence [13,14], but Meiklejohn et al. [10] make no mention of exercising precautions in this regard.*” Actually, Meiklejohn et al. did exercise some of the precautions identified in Figure 2, but these might have been overlooked by the authors of this paper as Meiklejohn et al referenced their original paper for the majority of their methods (Meiklejohn et al 2018, International Journal of Legal Medicine). Perhaps Meiklejohn et al should have rearticulated these precautions in their 2019 paper, but they did include some such as negative controls, bleach specimen wash prior to extraction, and amplification using various primer pairs. The authors need to modify the text either to state which precautions were taken by Meiklejohn et al, or remove the text ‘*make no mention of exercising precautions in this regard*’.

**6) Line 98-103:** Authors briefly discussed how even at a well curated museum there is a potential for taxonomic misidentification, particularly with older samples (see Line 102, “*As such, specimens may be misidentified, mirroring the situation reported in other studies*”). This reviewer acknowledges that this can be true, however it would be unreasonable for Meiklejohn et al. to second-guess the taxonomic classification given to a specimen at a museum by a subject matter expert. To confirm the authors’ hypothesis, they could have rechecked the identity of the vouchered specimen. Since it is standard practice to return unused specimen material to the repository, it would have been straight-forward for the authors to confirm their claims of misidentification by examination of the returned specimens. Since the authors did not report doing this examination, their assumptions are overreaching.

**7) Line 108:** Authors wrote, “*Importantly, all four cases of apparent misidentification reported by Meiklejohn et al. [10] involve species whose recognition is not straightforward*.” Upon review, Meiklejohn et al reported that common insect species were selected for inclusion and discussed that possible misidentifications (*i.e*., sister species) might have impacted identification success. However, if the species were incorrectly identified as the sister species, correct genus level identification still would have been obtained (this was not the case for four taxa in question). Thus, the authors need to update the text to accurately reflect that the type of species included in Meiklejohn et al were common ones.

**8) Line 118:** Authors state “*the supposed specimen..analyzed by Meiklejohn et al is almost certainly P. irritans*”. This reviewer does not know how the authors could possibly make this bold claim without having access to the specimen used in Meiklejohn et al to confirm the identity morphologically. Moreover, as stated in the above comment, it would be inappropriate to second guess the identification as outlined above. In retrospect, this supports the claim made by Meiklejohn et al. that care must be taken when using barcoding as the sole source for identifying material found in the environment, due to a wide variety of sources for possible misidentifications including morphological misidentification in barcode and genetic databases.

**9) Line 125-127:** Authors argued that these specific instances should be viewed as “*unconfirmed*”. However, these authors are leading entomologists. One could argue how would non-entomologists (such as Meiklejohn et al.) differentiate between an unconfirmed and a misidentification? Authors themselves mention in Line 36-39 that DNA based approaches to species identification can help fill the lack of taxonomic expertise that often impedes analysis. It is apparent from reviewing the manuscript that the purpose of the Meiklejohn et al. paper was different, as they were focussed on the general accuracy of GenBank and BOLD for returning a match to the expected species, not providing guidance on how matches should be reported out. Thus, the authors need to either remove the term ‘unconfirmed’ or explain how a user of bold with no entomology expertise could differentiate between an ‘unconfirmed’ and a ‘misidentification’.

**10) Line 128-149:** This reviewer agrees that identification of biological materials for forensics should be tackled interdisciplinary and with common sense, by looking at geographic information, etc. However, it is incorrect to suggest that Meiklejohn et al. scope was discussing forensics and DNA barcodes as their paper was solely on the utility of the DNA barcoding databases using a subset of curated museum species. It is unjust to assume the co-authors of Meiklejohn et al wrote in terms of forensics because of their affiliation. Presumably, Meiklejohn et al. would have been more specific regarding cautions if their objective was for forensic application. However, this was not the case. The authors need to modify the text to reflect the inaccuracy.

**11) Line 160-166:** Authors briefly summarized how it is possible to use GPS coordinate metadata included in BOLD to better source and identify samples. This is a reasonable suggestion for those who wish to more accurately identify a species for purely taxonomic reasons. However, doing so would require subject expertise in entomology or arthropod evolution to properly utilize this type of data (*e.g.,* specimen images and GPS coordinates) which is not reasonable to expect for most users of BOLD whose background knowledge will vary wildly from one user to the next. Finally, this kind of analysis was outside the scope of work performed by Meiklejohn et al. and therefore an unreasonable criticism of their methodology. This section either needs to be removed, or placed within context of the goal/purpose of the Meiklejohn et al study.

**12) Line 168-171:** This statement overstates that 11 species did not escape operational errors. The sequences for only 3 species were able to be fully resolved, but the sequences for the other 8 species were not. Authors failed to meet burden of proof as well as contradicted themselves throughout the paper writing that these species were “not as straightforward” and should be viewed as “unconfirmed”.

**Minor Technical Comments:**

**1) Line 55-59:** The impression the authors made here is that Meiklejohn et al used old specimens and thus this contributed to their misidentification results. Meiklejohn et al. included the age of the specimens as metadata and highlighted that amplicon/sequence length recovery was not correlated with specimen age. Thus, the authors need to modify the text to include this fact.

**2) Line 80**: Awkward wording of sentence. “Both BOLD and GenBank delivered generic identifications deemed incorrect for four specimens.” Not sure what this sentence means.

**3) Line 155-158:** Authors explain how BOLD and GenBank are similar. This is also mentioned in Meiklejohn et al and could have ben acknowledged.

**4) Line 175-178:** Authors suggest that Meiklejohn et al. did not use proper controls and quality checks; however, they did this according to their protocols that was cited in Methods. Meiklejohn et al out-referenced these steps from their earlier paper (Meiklejohn et al 2018 International Journal of Legal Medicine). The authors need to update the text to reflect this.

**Comments on Tables and Figures:**

**Table 1:** When checking the matches returned from the sequences listed in Table 1, it is apparent the authors mislabeled the query sequences. For example, upon downloading MK905402 from GenBank and searching against BOLD one returns a match for *Forifucla auricularia*. Thus, the authors need to place the red results from Meiklejohn et al under the MK905402/RC row for BOLD_all, not the MK905402 row alone.

Lastly, this table shows that 7 of 11 needed to be RC. However, only 3 species were able to be resolved. All 7 sequences that were not RC (as Meiklejohn et al. stated in their Discussion) were able to be confidently known to be incorrect due to their similarity statistic (<60%). Furthermore, the sequence orientation for BOLD was not explicated stated at the time Meiklejohn et al. queried the sequences and seems to only be affecting insect sequences (the BOLD fungal and plant algorithm was not sensitive to sequence orientation).

**Table 2:** What is N.D? the table description states “three categories of operational errors which compromised efforts by Meiklejohn et al…” Given this reviewer has concerns with the claims (and contradictions) the authors made about contamination, the ‘contamination’ column in this table needs to be removed. For instance, in line 85-87, the authors admitted that “*the other three mismatches.. along the 17 tested species ruling out internal contamination.*” Therefore, these species should not be included in the list as “contaminated” in the table.

Moreover, it is unclear what comprises the third operational error. Is it incorrect identification? Incorrect identification is not due to Meiklejohn et al. as these authors also had the same results. Neither Meiklejohn et al nor the authors performed taxonomic identification of the specimens. As presented, it appears to this reviewer that the authors wanted to find any reason for misidentification, aside from possible issues with BOLD. The column title needs to be modified accordingly.

**Figure 2:** This is a great figure to show proper workflow. Meiklejohn et al. followed many of these features (e.g., negative controls, quality assessment, etc.). Also given their sequences were submitted to GenBank and are from a coding region, the sequences would also have been checked for premature stop codons, indels, etc (this is part of GenBank’s QC process before acceptance).

**Additional Comments:**

**Appropriateness of the Methods**

There has been a shift in the guidelines for using BOLD for COI identifications – the interface now states that sequences must be submitted in forward orientation. The authors need to highlight that they searched the BOLD when this requirement had been articulated.

To support the claims made in this paper specifically on misidentification and cross contamination, the authors should have gone back to the pinned vouchered material at USNM to a) taxonomically confirm the species (addressing misidentification errors), and b) independently resequencing the questionable specimens (addressing cross-contamination issues). The addition of these steps would provide the authors evidence to classify the errors reported by Meiklejohn et al. A description of this work was not given.

**Support of the Conclusions by the Results**

The authors identified three reasons for the misidentification reported in Meiklejohn et al: 1) reverse complement sequences were searched against BOLD, 2) sequences were cross-contaminated, and 3) taxa included were either misidentified or part of a species complex. The authors results supported conclusion 1), however only circumstantial evidence/results wre provided to support conclusions 2 and 3.

**Over reaching Conclusions**

Several conclusions appear overreaching, especially those on misidentifications and cross-contamination. To validate these conclusions as previously stated, the authors should have gone back to the pinned vouchered material at USNM repository to a) taxonomically confirm the species (validating whether the specimens initially tested were indeed misidentified), and b) independently resequencing from questionable specimens (addressing cross-contamination issues).
