# Supplementary material for: BOLD and GenBank revisited – Do identification errors arise in the lab or in the sequence libraries?
Source: PLoS One. 2020 Apr 16;15(4):e0231814. doi: 10.1371/journal.pone.0231814 (PMC7162515; doi:10.1371/journal.pone.0231814)
Supplement: Attachment — Submitted filename: PONE-D-19-20600R1_Comments to Editor.docx [file pone.0231814.s005.docx]

**Comments to the Editor for PONE-D-19-20600R1:**

Based on statements made by authors in lines 24-26, as well as the majority of the authors’ discussion points, it is this reviewer’s opinion that this article is trying to blend a criticism of Meiklejohn et al. methodology along with providing suggestions for mitigating potential misidentifications in future studies. Based on all of the above comments, I do not believe the burden of proof has been met to back the claims made by the authors regarding the sources of misidentification in Meiklejohn et al. The authors need to make major revisions to this paper to address the shortcomings identified in my review, prior to acceptance for publication. Further, it should be clearly noted that Meiklejohn et al.’s paper did not discuss forensics as it was outside the scope of their paper. It is unjust to assume that they are speaking in terms of forensics just because of their affiliation. If their objective was for forensic application, Meiklejohn et al. would have been more specific in regards to cautions, workflow and interpretation guidelines. However, this was neither the intended audience nor focus of their paper. Authors’ paper should not have the title “forensics and DNA barcodes” as this is not a true representation of the Meiklejohn et al paper.

It is important to disclose that I am the corresponding author for the Meiklejohn et al paper that is being critiqued by Pentinsaari et al. This put me in a unique position such that I have a more detailed knowledge and understanding of the work completed by Meiklejohn et al, along with the purpose and application. However, I have constructed my review in such a way that only information that would be available to a reader of Meiklejohn et al has been highlighted and discussed. I have been professional and respectful, as suggested in the PLOS Peer Review Checklist and I included positive feedback as well. I hope that my suggestions for improvement of the manuscript are accepted by the authors, in case a revision is requested.
